# VO_2_ metasurface smart thermal emitter with high visual transparency for passive radiative cooling regulation in space and terrestrial applications

**DOI:** 10.1515/nanoph-2022-0020

**Published:** 2022-04-25

**Authors:** Kai Sun, Wei Xiao, Callum Wheeler, Mirko Simeoni, Alessandro Urbani, Matteo Gaspari, Sandro Mengali, C.H. (Kees) de Groot, Otto L. Muskens

**Affiliations:** Physics and Astronomy, Faculty of Physical Sciences and Engineering, University of Southampton, Southampton SO17 1BJ, UK; Electronics and Computer Science, Faculty of Physical Sciences and Engineering, University of Southampton, Southampton SO17 1BJ, UK; Consorzio CREO, SS.17 Località Boschetto, L’Aquila 1-67100, Italy

**Keywords:** metasurfaces, plasmonics, radiative cooling, thermochromic, vanadium dioxide, VO_2_

## Abstract

Smart radiative cooling devices based on thermochromic materials such as vanadium dioxide (VO_2_) are of practical interest for temperature regulation and artificial homeostasis, i.e., maintaining stable equilibrium conditions for survival, both in terrestrial and space applications. In traditional solar reflector configurations, solar absorption in the VO_2_ layer is a performance limiting factor due to the multiple reflections of sunlight in the stack. Here, we demonstrate a visually transparent, smart radiator panel with reduced solar absorption. An Al-doped ZnO transparent conducting oxide layer acts as a frequency selective infrared back-reflector with high transmission of solar radiation. In this study we make use of high-quality VO_2_ thin films deposited using atomic layer deposition and optimized annealing process. Patterning of the VO_2_ layer into a metasurface results in a further reduction of the solar absorption parameter *α* to around 0.3, while exhibiting a thermal emissivity contrast Δ*ε* of 0.26 by exploiting plasmonic enhancement effects. The VO_2_ metasurface provides a visual spectrum transmission of up to 62%, which is of interest for a range of applications requiring visual transparency. The transparent smart metasurface thermal emitter offers a new approach for thermal management in both space and terrestrial radiative cooling scenarios.

## 1 Introduction

Radiative cooling coatings are receiving great interest for their potential as environmentally friendly and energy saving solution for cooling of buildings, personal thermal management, energy harvesting [1], [2], [3], [4], [5], [6], and improvement of solar cell performance [7, 8]. Terrestrial applications are targeted using polymer coatings which can be manufactured at low cost and over large areas. In contrast, optical solar reflectors (OSRs) used in spacecraft need to be durable, inorganic coatings able to withstand the extreme conditions of the space environment. OSRs in form of thin glass tiles coated with a reflecting metallic film have been used for decades as critical parts in the thermal control system of spacecraft, to dissipate heat and maintain survivable operational temperatures for on-board instruments and crew [9, 10]. OSR tiles pose challenges related to the assembly, integration and testing (AIT) onto spacecraft and add significant mass to the mission, hence reducing the payload capacity and increasing launch costs.

Recently, thin-film OSR technologies have been proposed as an alternative solution that could offer advantages in form-factor, flexibility of integration and reduced cost, arguments that are of particular interest for small spacecraft, such as CubeSats, micro- and nano-satellites. The first generation of flexible first-surface OSR coatings was developed based on an interferential ceramic metallic (CERMET) multilayer stack, which is now approaching commercializations [11]. Apart from multilayer filter stacks of many micrometers in thickness, the use of in-plane patterning of layers in combination with plasmonic resonance effects could be used to achieve micrometer thin devices. Metasurface OSRs based on transparent conductive oxides (TCOs) have been recently reported for their broadband infrared (IR) emissivity corresponding to surface plasmon resonances of the metasurface [12], [13], [14].

Radiative cooling applications based on a constant high emissivity emitter have the disadvantage of continuously dissipating heat, even when operating in a start-up phase, during night-time, or when in sleep or safe mode. In a number of applications, temperature regulation within a certain range is desired to stabilize the systems against varying conditions. Temperature regulation through emissivity control can be done either through active louver systems [15], mechanically deployable panels [16], [17], [18], bio-inspired bi-morph materials [19] or by passive systems based on thermochromic materials [14, 20]. A variety of designs using thermochromic VO_2_ thin films have been reported in literature [21], [22], [23], [24], [25], [26], [27]. Most reported radiative cooling solutions are based on metallic reflector structures for high reflection in the visible spectrum [5, 13, 28, 29]. Recently, visually transparent radiative cooling solutions have been proposed through film stack structures to achieve a high IR emissivity for example in solar cell applications [30], [31], [32]. These are all static, i.e., nonvariable emissivity, applications. Efforts in achieving smart windows so far have been mainly directed toward tuning the visual transmission (tinting) and blocking of the near-IR part of solar spectrum for reduced solar heating of interiors [33], [34], [35], [36], [37], [38]. Thermochromic smart windows for radiation cooling have only very recently been demonstrated using VO_2_ nanoparticles in a polymer matrix [39].

Here, we propose a metasurface radiative cooling device that combines variable thermal emissivity and high optical transparency in the solar spectrum. The IR thermal emitter is based on a metamaterial perfect absorber (MPA) design [26]. The IR tunable emissivity is achieved by controlling the absorption strength of the thermochromic material, VO_2_, by tuning the material across the insulator-to-metal transition (IMT). High visual transparency is achieved by replacing the conventional metal back reflector by a layer of Al-doped ZnO (AZO). The TCO characteristics of the AZO ensure functionality as a frequency-selective back-reflector where radiation at wavelengths longer than 3 µm is effectively reflected, while high visual transmission is simultaneously achieved in the visible spectrum. This modified design offers the advantage that only a single pass through the VO_2_ is required in the visible range, hence significantly reducing the solar absorption. Sunlight essentially passes through the panel and is transmitted with low energy absorption, thus fulfilling a similar requirement as conventional OSR panels where sunlight is reflected.

Compared to a thin-film stack, the visual transparency of the device is further improved by structuring the VO_2_ layer into a metasurface with VO_2_ coverage of around 50% [26]. Our study combines numerical device modeling and an experimental demonstration of the principle of operation. As a first step, the metasurface design is optimized using FDTD numerical simulations. In our experimental work, we present a VO_2_ metasurface obtained using a highly controlled atomic layer deposition (ALD) process. We achieve VO_2_-based metasurface thermal emitters with a solar transmission of 58%, reflectivity of 20% and solar absorption parameter *α* of 0.32. An IR emissivity tunability Δ*ε* of 0.26, tuning the emissivity from 0.55 to 0.81 is obtained, which we show is limited by emissivity of the TCO back-reflector. Finally, we numerically evaluate the performance in terrestrial radiative cooling applications.

As the structured metasurface design offers an improved visual transmittance of 62%, this approach opens perspectives on new device concepts in space, which can also be extended to potential terrestrial applications as radiation coolers for smart windows, LCD display screens and solar panels.

## 2 Device design and numerical modeling

**Figure 1:**
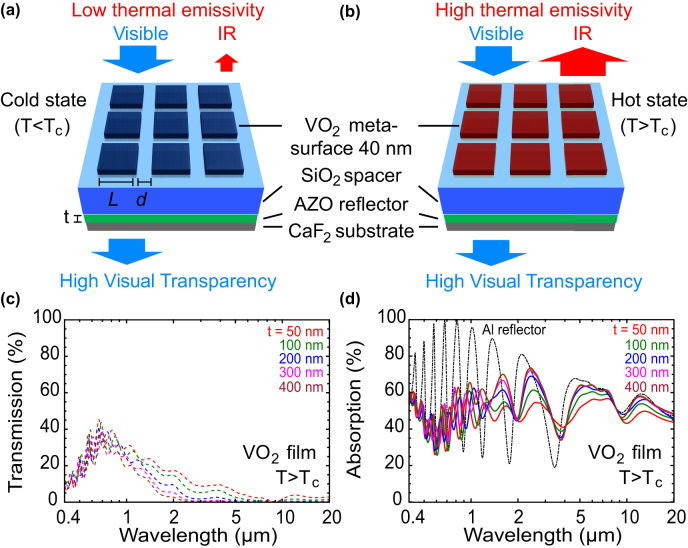
Operation of smart VO_2_ metasurface thermal emitter. (a) Cold state schematic (b) hot state schematic (c and d) numerical simulations of transmission (dashed lines, c) and absorption spectra (solid lines, d) for VO_2_ thin film thermal emitter in the hot state (*T* > *T*_C_) with AZO reflector thickness *t* ranging from 50 to 500 nm. Results for Al-reflector design are shown by dash-dotted line in d for comparison.

The operation of the smart VO_2_ thermal emitters is shown in Figure 1. It illustrates the schematic layout of the device and its behavior at temperature below the IMT (cold state, Figure 1a) and above the IMT (hot state, Figure 1b). VO_2_ is a thermochromic material with a critical temperature *T*_C_ of around 68 °C [40, 41]. Below the transition range around *T*_C_, VO_2_ is entirely in the monoclinic structure (VO_2_ (M1)) and behaves as dielectric in the IR region. Above *T*_C_, VO_2_ is in the tetragonal rutile structure (VO_2_ (R)) and behaves as a metal in the IR region.

The stack design is based on a Salisbury screen structure as schematically shown in Figure 1c [42]. The three-layer stack consists of the back-reflector, the dielectric spacer and the VO_2_ layer. The SiO_2_ spacer thickness of 1200 nm results in constructive interference of the incident and reflected thermal radiation around 10 µm wavelength at the metasurface, maximizing the absorption in the VO_2_ layer.

Unlike radiation coolers [28, 43] or optical solar reflectors [13, 26], we use a frequency selective IR reflector made by an Al-doped ZnO (AZO) film instead of a broadband metallic reflector such as aluminium. For the AZO carrier density of 5.8 × 10^20^ cm^−3^ used in our study, the AZO layer is metallic in the near and far infrared (NIR/FIR) range but dielectric in the visible/near infrared (NIR) range from 400 nm to around 2000 nm wavelength [44]. Figure 1d shows the calculated transmission and absorption response of a stack using an unpatterned VO_2_ thin-film as function of the AZO thickness for the hot state. For AZO thickness t > 300 nm the reflection saturates, resulting in an IR absorption in the 2.5–20 µm band which is independent of thickness. The behavior of the VO_2_ stack in the NIR/FIR is identical to a stack in which the AZO is replaced by a 100 nm Al back reflector. The difference though is in the visible and NIR range, where the AZO stack achieves significant transmission/transparency.

**Figure 2:**
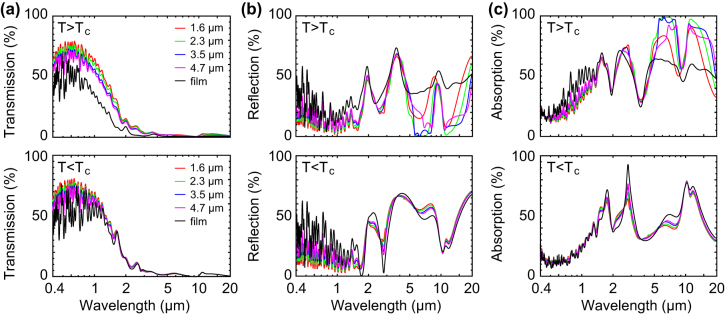
Numerical simulations of optical spectra of VO_2_ metasurface thermal emitters with different VO_2_ feature size *L* and gap *d* = 2 µm. (a–c) Transmission, reflection and absorption in the hot state (*T* > *T*_C_) and cold state (*T* < *T*_C_). Simulations for unpatterned thin film are shown for comparison.

The VO_2_ is patterned into a metasurface consisting of an array of square features tuned for achieving a broadband resonance covering the thermal blackbody spectrum [26]. To explore the optimized design structure, numerical simulations were used to investigate design for high visual transparency and desirable IR emissivity. The AZO reflector thickness has been fixed at *t* = 300 nm based upon results presented in the previous paragraph. Figure 2 shows simulated spectra of VO_2_ metasurfaces with different feature sizes at a fixed gap for both hot and cold state. In term of transmission in the hot state, all devices give near zero transmission above 3 µm, indicating the metallic behavior of the AZO film which fully reflects IR spectra. The metasurface devices give higher visual transparency than the planar film device, with the smaller feature size giving slightly higher visual transmission owing to its lower VO_2_ coverage ratio. Importantly, the absorption spectrum in the hot state shows a significant increase in absorption in the IR range between 5 and 20 µm for the metasurface as compared to the planar film. The improvement in both visual transparency and IR absorption emissivity shows the clear advantage of patterning the VO_2_ film to create a metasurface. In the cold state, the metasurface devices still have a superior visual transparency although the IR behavior is now nearly identical to the planar thin-film stack as there is no confinement if the VO_2_ is not in the metallic state. Angle dependent simulations are presented in Supplementary Materials Figure S5 for one of the metasurface designs. The IR emissivity contrast decreases from 0.3 to 0.2 with incident angle increasing from 0 to 60°. With the same incident angle increase, the solar absorption (*α*_hot_) and visual transmittance (*T*_vis,hot_) only slightly changes by a decrease from 0.34 to 0.30 and an increase from 0.57 to 0.64, respectively.

**Figure 3:**
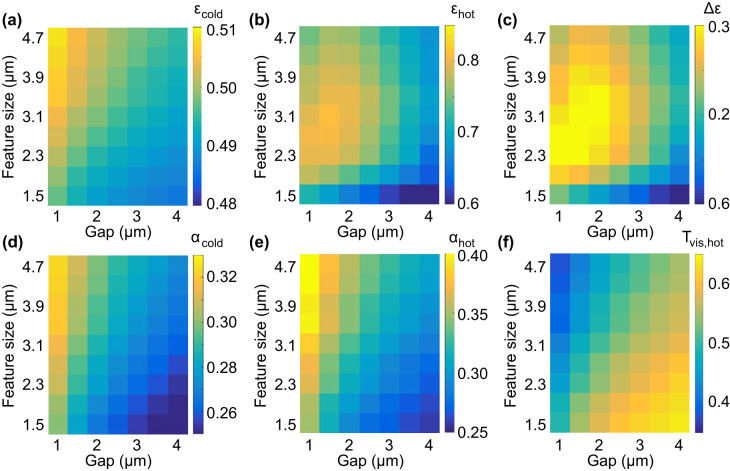
Design optimization as 2D sweep of VO_2_ square feature size *L* and gap *d* through numerical simulations. (a) IR emissivity in cold state *ɛ*_cold_, (b) IR emissivity in hot state *ɛ*_hot_, (c) IR emissivity contrast Δ*ɛ*, (d) solar absorption in cold state *α*_cold_ (e) solar absorption in hot state *α*_hot_. (f) Visual transmission in the hot state, *T*_vis, hot_. Calculations based on space environment.

The enhanced absorption in the hot state is a result of plasmonic effects in the nanostructured metasurface. The plasmonic resonance effect allows maintaining the same *ε*_hot_ as for the continuous thin film while reducing the VO_2_ coverage by more than twofold trough metasurface patterning. This effect is most clearly seen in Figure 2c where the absorption of the metasurfaces exceeds that of the unpatterned film in the spectral range from 6–18 µm. The resonant nature of this effect can also most clearly be seen when studying *ε*_hot_ and the tunability Δ*ε*, as function of both metasurface feature size and gap width as is shown in Figure 3. Figure 3 shows the investigation of device performance for varying feature size (*L*) and gap size (*d*) obtained using numerical simulations. The thermo-optic performance of metasurface can be defined in terms of solar absorption *α* and IR emissivity contrast (Δ*ɛ*). The solar absorption *α* is calculated by the transmission weighted with solar radiation between 0.4 and 2.5 µm using black body spectra at 5777 K (space or above Ozone), as is typical for solar cells [45]. IR emissivity *ɛ* is calculated over the IR range between 2.5 and 20 µm [26].

Figure 3a–c shows color map of calculated emissivity as function of feature size and gap size. At low temperature, IR emissivity is little affected by feature size and gap size and consistently around 0.5. Hot IR emissivity *ɛ*_hot_ has a strong dependence and emissivity peaks at 0.81 for *L* = 3.1 µm and *d* = 1.5 µm. The corresponding IR emissivity contrast Δ*ɛ* = 0.3. The solar absorption in both states is shown in Figure 3d and e. At both low and high temperatures, the solar absorption *α* decreases with decreasing feature size and increasing gap size owing to VO_2_ coverage ratio decrease, as previously discussed. We also evaluate in Figure 3f the visual transparency, *T*_vis_, in the hot state by weighting the visible transmission with the photopic sensitivity function of the eye, as discussed in more detail further below in Section 3.2. Visual transparency is strongly related to the *α* parameter and generally improves with lower VO_2_ area coverage. A balance of high IR emissivity contrast (Δ*ɛ*), moderate solar absorption and good visual transparency is obtained over a range of sizes around 2–4 µm and 2 µm gap, which is the range chosen for our device fabrication.

## 3 Experiments

### 3.1 Atomic layer deposition of the VO_2_ thermochromic layer and unpatterned thin-film results

**Figure 4:**
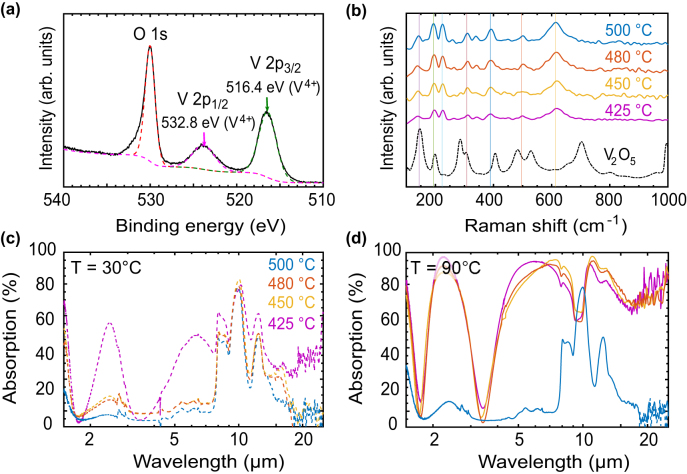
VO_2_ by atomic layer deposition process and the characterizations after different post-anneal treatments. (a) X-ray photoelectron spectroscopy (XPS) result showing key energies corresponding to the VO_2_ phase. (b) Raman spectra of VO_2_ with 1 h anneal at temperatures of 425 °C, 450 °C, 480 °C and 500 °C. (c and d) FTIR absorption spectra of VO_2_/SiO_2_/Al stack measured at 20 °C (dashed lines, c) and 90 °C (solid lines, d) for the annealed samples of (b).

For the proposed smart VO_2_ metasurfaces, the thermochromic property of VO_2_ is the most critical part. A process based on the atomic layer deposition (ALD) technique was developed and optimized to achieve desirable optical properties, accurate thickness control and large-substrate uniformity (see Supplementary Materials S1 and S5). The vanadium precursor, [V(NEtMe)_4_ (TEMAV), from Strem Chemicals], was heated up to 85 °C to achieve a sufficient vapor pressure. Recently, VO_2_ processes based on TEMAV were demonstrated by a number of research groups using as oxygen precursor ozone [46], [47], [48], [49] or water [50], [51], [52]. In this work, water was chosen as oxygen precursor as the more promising route to stoichiometric VO_2_. Figure 4a provides a summary of optimized key process parameters and growth rate and uniformity. The deposition temperature was set as 150 °C as the precursor risks thermal decomposition above 200 °C. The growth rate is extracted to be 0.37 Å/cycle and 6-inch substrate uniformity is below 2%. The growth rate is lower than some reported ozone process of ∼0.8 Å/cycle [47, 49, 53]. However, there is also some inconsistency in reported growth rates [50, 54]. Therefore, the excellent uniformity of below 2% over 6-inch substrate indicates that the developed process is a self-limiting ALD growth (as the ALD tool, unlike CVD tools, is not hardware optimized for uniformity).

A 40 nm VO_2_ film as deposited was investigated by X-ray photoelectron spectroscopy (XPS), the binding spectrum is shown in Figure 4a. No argon etch was performed as argon etch was known to preferentially etch oxygen atoms, which then affects the vanadium oxide stoichiometry with extra oxidation states [55]. The binding energies values were charge corrected to the O1*s* peak at 530 eV due to O–V bonds [56]. The V2*p* energy level splits into the V2*p*_1/2_ and V2*p*_3/2_ components due to orbital splitting at 523.8 eV and 516.4, respectively, and these values are consistent with those reported for VO_2_ [55, 57]. Therefore, XPS analysis shows as-deposited film is mainly VO_2_ with V present as V^4+^ and similar result was also seen in the work by Peter et al. [51]

As the VO_2_ film is amorphous as deposited, a post-anneal treatment is required to crystalize the VO_2_ into monoclinic VO_2_ (M1) for thermochromic properties. Based on literature knowledge [54], the anneal condition is a trade-off between temperature, oxygen pressure and anneal time. For the anneal studies, VO_2_ was deposited on an VO_2_/SiO_2_/Al OSR stack on 6-inch Si substrate to generate a sufficiently large number of identical samples for anneal test at various conditions. Also the anneal conditions are more easily compared for a high IR emissivity contrast. We tested the performance of VO_2_ films annealed in O_2_ pressure of 40 mTorr for 1 h, for different temperatures of 425 °C, 450 °C, 480 °C and 500 °C. Figure 4b shows Raman spectra of the annealed VO_2_ films with a bulk V_2_O_5_ spectrum shown as a reference. Known Raman peaks for VO_2_ (M1) were also marked at 144 cm^−1^, 193 cm^−1^, 223 cm^−1^, 308 cm^−1^, 389 cm^−1^, 497 cm^−1^ and 613 cm^−1^ [57], [58], [59]. All four VO_2_ films are seen to exhibit Raman peaks highly consistent with known VO_2_(M1) peaks and none matches V_2_O_5_ peaks, indicating that all four anneal conditions achieve VO_2_(M1) with little presence of V_2_O_5_.

IR absorption spectra are presented in Figure 4c and d for VO_2_/SiO_2_/Al film reflectors using Fourier transform infrared (FTIR) measured respectively at 20 °C (c, denoted as cold) and 90 °C (d, denoted as hot). In the cold state, VO_2_ devices annealed at 450 °C, 480 °C and 500 °C shows only little absorption over a broad IR range except for a band of absorption peaks around 10 µm due to the vibrational modes of the underlying SiO_2_ layer. In comparison, a significant higher IR absorption is seen for the VO_2_ film annealed at 425 °C (purple line in Figure 4c). The low IR absorption in the cold state is attributed to the dielectric properties of VO_2_ [26]. In the hot state, VO_2_ devices annealed at 425 °C, 450 °C and 480 °C show a broad IR absorption whilst the material annealed at 500 °C gives little IR absorption. The high IR absorption is attributed to the metallic property of VO_2_ in the hot state [26]. We see that annealing in the temperature range of 450 °C–480 °C result in a desirably high IR contrast. The huge difference in the IR optical properties observed for these different anneal conditions is of great interest as these films are all identified as VO_2_ by Raman spectroscopy. Thus, the VO_2_ anneal condition needs to be particularly well optimized for this application. After material optimization, the VO_2_ using the promising anneal condition was successfully applied to stacks grown on CaF_2_ substrates.

### 3.2 Fabrication and characterization of visually transparent VO_2_ smart metasurface thermal emitters

**Figure 5:**
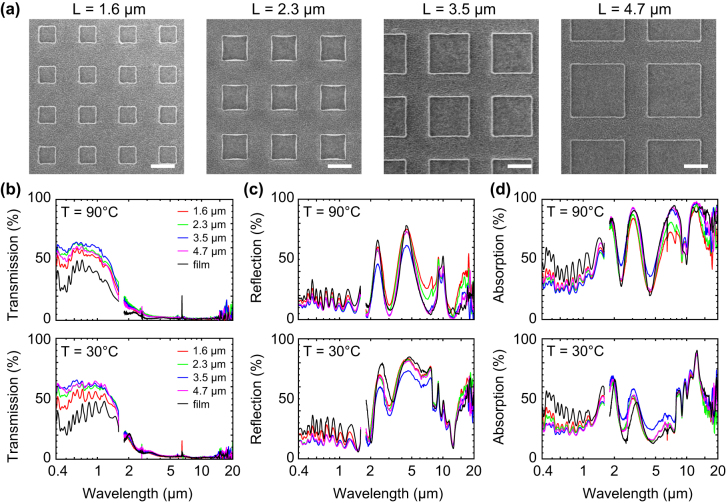
SEM images and experimental optical spectra of VO_2_ metasurfaces as a function of feature size. (a) SEM images for metasurfaces with gap size fixed at *d* = 2 µm and feature sizes *L* = 1.5 µm, 2.3 µm, 3.5 µm and 4.7 µm. (b–d) Transmission, reflection and absorption spectra at 30 °C and 90 °C, for the VO_2_ metasurfaces with four different feature sizes (colored lines) as well as the unpatterned thin film device (black line).

To study the behavior of the transparent metasurface thermal emitters, we fabricated and evaluated a device stack on a 1 mm thick CaF_2_ substrate. Figure 5a shows SEM images of the VO_2_ metasurface with feature size *L* of 1.5 µm, 2.3 µm, 3.5 µm and 4.7 µm. For all three sizes, VO_2_ features are well defined in square shape and the gaps are accurately defined as *d* = 2 µm. Optical spectra of their metasurfaces and film are presented in Figure 5d–i for the spectral range from 0.4 to 20 µm covering visible, NIR and IR, measured at 90 °C (hot state, d–f) and at 30 °C (cold state, g–i). The absorption (*A*) is obtained from the transmission (*T*) and reflection (*R*) using the relation *A* = 1 − *R* − *T*. In our experiments we use a FTIR microscope with numerical aperture of 0.58, corresponding to a range of angles from 0°–35°.

The VO_2_ metasurfaces provide up to 60% transmission in the visible range and almost zero transmission in the thermal IR range (>2.5 µm) in both the hot and cold states (Figure 5d and g). The CaF_2_ substrate is completely transparent for wavelengths <10 µm and we find that the transmission of the bare metasurface stack in this range is in good agreement with the numerical simulations of Figure 2. Compared with the planar film device (black curve), metasurfaces offer significantly improved visible/NIR transmission which is related to the reduced VO_2_ coverage in the metasurface geometry.

In reflection (Figure 5e and h), VO_2_ metasurfaces result in a weakly oscillating reflection around 20% in the spectral range of 0.4–1.5 µm. In the mid-IR range between 2 and 8 µm wavelength, the cold state shows an overall high reflectivity which is associated with the AZO back-reflector. In the hot state, stronger oscillations are seen with an amplitude depending on the metasurface feature size, which is caused by an interplay of the Fabry–Perot fringes in the dielectric spacer and the strong absorption in the VO_2_. At odd multiples of a ¼ wavelength, a minimum reflection is seen as the antinodes coincide with the VO_2_ layer (Salisbury screen effect), while for even multiples nodes at the location of the VO_2_ result in maxima in the reflection.

The absorption spectra (Figure 5f and i) show a relatively flat absorption in the visible and NIR range around 30–35%, where a lower visible absorption is found for the metasurface geometry than for a planar film. Strong IR absorption is seen in the hot state (Figure 5f) both for the planar film as well as for the larger feature metasurfaces, while the IR absorption is reduced for smaller feature sizes. The IR metasurface absorption is influenced both by plasmonic resonance effects and VO_2_ area coverage and a balance of both is needed as clearly illustrated by the simulated parameter maps of Figure 3. In the cold state, the IR absorption between 10 and 20 µm (and associated dip in reflectivity) is related to vibrational (LO and TO) modes of the SiO_2_ dielectric spacer.

### 3.3 Performance parameters of visually transparent VO_2_ smart metasurface thermal emitters

The experiments of Section 3.2 show that the VO_2_ metasurface achieves an improved visible/NIR transparency in both the cold and hot states and an IR emissivity contrast equivalent to the planar film. Similar to their counterparts with Al reflector, the IR absorption enhancement is attributed to the plasmonic effects of the VO_2_ metasurface [26]. Table 1 summarizes the obtained values of IR emissivity in term of *ɛ*_hot_, *ɛ*_cold_ and emissivity contrast Δ*ɛ*, as well as the solar absorption parameters *α*_hot_ and *α*_cold_. For IR emissivity and solar absorption calculation, details are available in our previous works [13, 26]. Additionally, visual transmittance (*T*_vis, hot_ and *T*_vis, cold_) in the hot and cold states in the human visual range (*T*_vis_) is obtained from the spectra using the equation [60]:

(1) $$T_{\text{vis}}=\int T\left(\lambda \right)\Phi_{\text{lum}}\text{d}\lambda /\int \Phi_{\text{lum}}\text{d}\lambda$$

where T(λ) is the measured transmittance and $\Phi_{\text{lum}}$ is the standard luminous efficiency function for photopic vision in the wavelength range of 400–780 nm [61].

**Table 1:** Summary of calculated IR emissivity, solar absorption and visual transmittance at hot and cold status using measured experimental spectra (Figure 5).

*L* (µm)	*ɛ*_hot_	*ɛ*_cold_	Δ*ɛ*	*α*_hot_	*α*_cold_	*T*_vis, hot_	*T*_vis, cold_
1.5	0.69	0.51	0.18	0.36	0.33	49%	51%
2.3	0.74	0.52	0.22	0.33	0.30	57%	59%
3.5	0.82	0.58	0.24	0.33	0.28	62%	60%
4.7	0.81	0.54	0.26	0.36	0.29	58%	55%
Film	0.80	0.54	0.26	0.46	0.43	29%	35%

The thermal emissivity contrast Δ*ɛ* between hot and cold states is the critical performance metric for smart emitter devices and is given by the difference between hot and cold emissivity (*ɛ*_cold_ and *ɛ*_hot_) at 90 °C and 30 °C, respectively. For *ɛ*_hot_, the feature size is seen to have an impact due to the plasmonic resonance enhancement to the IR absorption. Larger feature sizes of 3.5 and 4.7 µm give similar *ɛ*_hot_ to the planar film whilst smaller feature size of 1.5 and 2.5 µm give smaller values of *ɛ*_hot_. For *ɛ*_cold_, metasurfaces are not very different from the planar film since the dielectric VO_2_ gives no plasmonic enhancement and the cold emissivity is mostly associated with the vibrational modes of the dielectric SiO_2_ spacer and the emissivity contribution of the AZO back-reflector. Overall, the emissivity contrast slightly increases with the feature size from 1.5 to 4.7 µm and peaks at Δ*ɛ* = 0.26 for a feature size of 4.7 µm. IR emissivity hysteresis is shown in Supplementary Materials Figure S3.

For solar absorption, all metasurfaces show significantly reduced absorption compared to the planar film in both cold and hot states. The metasurface with feature size of 3.5 µm gives the lowest value of *α*_cold_ = 0.33, which is 28% lower than the planar film (*α*_film, cold_ = 0.46). The hot-state solar absorption dips at *α*_hot_ = 0.28 also for the 3.5 µm feature size corresponding to a 35% improvement over the planar film (*α*_film, hot_ = 0.43). Therefore, VO_2_ metasurfaces with optimized design provide equivalent IR emissivity with a significantly reduced solar absorptions than the equivalent thin-film stack. This reduction in solar absorption is highly advantageous both for its improved thermal performance (as quantified by the ratio *ɛ*/α in either state) as well as for the strongly increased visual transparency which overcomes the serious issue of the lack of VO_2_ visual transparency even at the reduced thickness of 35 nm [45]. Direct comparison with conventional OSR stacks using a metal back-reflector show a further significant reduction in *α* owing to the difference in absorption in single-pass transmission through the VO_2_ film compared to double-pass reflection in a conventional OSR design [6].

Finally, we evaluate the visual transparency performance of these metasurfaces. All metasurfaces in the hot state give significantly improved visual transmittance over the planar film and the metasurface with feature size of 3.5 µm gives the highest value of *T*_vis, hot_ = 62%, which is a more than double the value of the corresponding planar thin-film stack (*T*_vis, hot_ = 29%). The same trend is also seen in the cold state where visual transmittance peaks at 60% for 3.5 µm feature over the planar film which is 71% improvement over the planar film.

## 4 Discussion

### 4.1 Transition from reflection to transparent OSR and the role of carrier density on the device performance

Compared with OSR designs based on VO_2_ metasurface stacks using an Al back-reflector [26], the emissivity contrast of 0.26 is considerably lower. To investigate this, the effect of AZO carrier density has been studied through numerical simulations between 1 × 10^20^ cm^−3^ and 1 × 10^22^ cm^−3^. The simulated spectra are presented in Supplementary Materials Figure S4.

**Figure 6:**
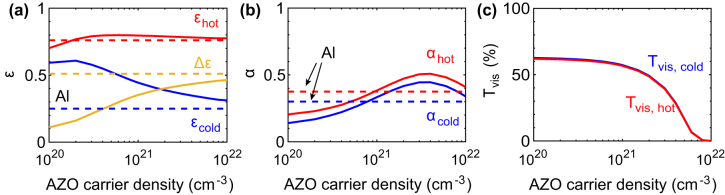
Effect of AZO carrier density on the VO_2_ metasurface performance. (a) IR emissivity, (b) solar absorption and (c) visual transmittance.

Figure 6 shows the simulated performance of the VO_2_ metasurface (*L* = 3.5 µm and *d* = 2 µm) with different AZO carrier densities together with that using an aluminium reflector presented as reference. Hot IR emissivity (*ɛ*_hot_) is little affected and consistent with that of the Al reflector design (dashed line, red). Unlike *ɛ*_hot_, *ɛ*_cold_ decreases with AZO carrier density when above 2 × 10^20^ cm^−3^. The emissivity contrast, as a combined effect, increases with AZO carrier density towards that of the Al reflector. The carrier density of fabricated AZO in this work is estimated to be around 6 × 10^20^ cm^−3^ and its emissivity contrast is consistent with the performance predicted by the simulation. The infrared emissivity tunability Δ*ɛ* is mainly affected by the emissivity of the backreflector itself which is higher for lower carrier density where the skin depth is large and electromagnetic fields are more strongly absorbed inside the conductor. With AZO carrier increasing, solar absorptions (Figure 6b) at hot and cold (*α*_hot_ and *α*_cold_) increase and peak at carrier density of around 4 × 10^21^ cm^−3^. This increase is related  to the closing of the visual transparency window in Figure 6c as the plasmon frequency shifts from the infrared, across the visible to the ultraviolet range. For even higher carrier densities, solar absorption losses are reduced as the electromagnetic skin depth reduces penetration of fields inside the lossy metal, eventually reaching the limit of the Al metallic backreflector (dashed lines), where Al is a Drude metal with carrier density of 1.8 × 10^23^ cm^–3^.

Thus, lower AZO carrier density is preferred for lower solar absorption. The visual transmittance (Figure 6c) maintains a value at around 60% over a wide range of low carrier densities, but rapidly decreases when the AZO carrier density exceeds 1 × 10^21^ cm^−3^. This decrease is attributed to the metallic response at shorter wavelengths at higher carrier density. Therefore, the AZO carrier density needs to be chosen as trade-off between emissivity contrast, solar absorption and visual transmittance. Further improvement of Δ*ε* may be possible by replacing the SiO_2_ with another low-emissivity material, such as CaF_2_, MgF_2_, or ZnS, to reduce the emissivity background of other parts of the stack.

Our experimental results demonstrate that VO_2_ metasurfaces with a transparent backreflector with carrier density around 6 × 10^21^ cm^−3^ offer superior visual transmittance over planar films whilst maintaining an equivalent thermal emissivity contrast, making them potentially superior candidates in see-through applications, e.g., smart windows [39, 60]. We point out that the VO_2_ in this work has a transition temperature around 68 °C, which is significantly above the desirable room temperature operation for many temperature regulation applications. However with introduction of dopants, such as tungsten, into VO_2_, the transition temperature of the VO_2_ can be reduced to room temperature [39, 62], [63], [64], [65].

### 4.2 Cooling power performance studies

Metasurface based technologies are still at a lower technology level compared to thin-film coatings which can be more easily scaled up. The small scale of current generation of metasurface device demonstrators prevents a direct validation through field studies. We emphasize that IR absorption measurements under the assumption of Kirchhoff’s law are an accepted standard in the characterization of thermal control coatings which is generally highly correlated with the thermal performance in existing literature. We therefore estimate the theoretically expected thermal performance on the basis of the experimentally derived parameters in order to evaluate the cooling power that can be theoretically achieved using our metasurface smart radiator devices (see also Supplementary Materials Section S5). We separately consider here applications in radiative cooling management both in terrestrial and space environments.

To quantify the cooling power for terrestrial applications, three contributing factors have to be considered. The (1) solar absorption *P*_sun_, (2) the thermal radiation from the atmosphere *P*_a_, and (3) the energy radiated from the device itself *P*_r_. The largest heating comes from the absorption of the incident solar flux, which has a total power of 987 W m^−2^ incident on the Earth’s surface. Here we use the value corresponds to the standard AM1.5 global standard to provide a meaningful comparison between different devices. More detailed evaluations based on specific geographic locations and daytime variation in solar irradiation should be made for specific applications [48].

Table 2 shows the simulated values for power per unit area in hot and cold states for varying feature sizes and for the planar film devices calculated using the standard radiative cooling equations [6, 66], [67], [68], [69]. The atmospheric effect is taken into account by considering the thermal radiation absorbed by the device from an environment at temperature of 300 K. The difference in *P*_a_ between cold and hot states is due to the difference in solar absorption of the VO_2_ below and above the critical transition. The net power flux *P*_r_ − *P*_a_ − *P*_sun_ is presented as a standard daytime radiative cooling performance figure showing the total net cooling capability under daytime solar illumination, while for comparison the night-time radiative cooling performance *P*_r_ − *P*_a_ is also indicated.

**Table 2:** Calculated terrestrial performance in radiation cooling and solar power through at room temperature for VO_2_ in cold (blue, 30 °C), and hot (orange, 90 °C) states.

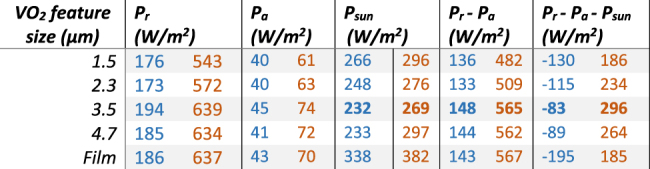

Calculations were also performed for space applications where radiation contributions by the atmosphere are negligible and the incident power from the sun is much greater, which can reach up to 1345 W m^−2^ for continuous direct exposure, while in-orbit the total sun hours depend on the inclination of the plane of orbit known as the β-angle. Typical results for full direct exposure in the space environment are presented in Table 3, again for VO_2_ in the cold and hot phases.

**Table 3:** Calculated performance for radiation cooling in the space environment, at room temperature for VO_2_ in cold (blue, 30 °C), and hot (orange, 90 °C) states.

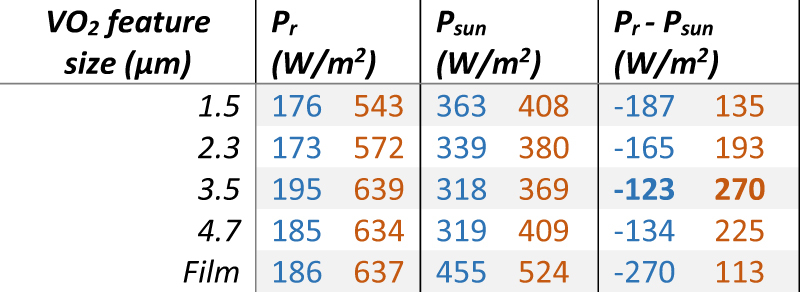

The net power *P*_r_ − *P*_a_ − *P*_sun_ radiated by the devices in a terrestrial environment reaches a maximum for feature sizes of 3.5 µm for both the cold and hot states. The 3.5 µm feature size appears to be the optimized value for IR terrestrial cooling with a hot state cooling power of 296 W m^−2^. The radiated power from the device itself, *P*_r_, increases by a factor of three between the cold and hot states, of which a factor two results from the fourth-power scaling of total radiation power with temperature, while a factor 1.5 is attributed to the thermochromic emissivity change Δ*ɛ*. We see that the optimized metasurface reflector outperforms the equivalent thin-film device by 60% in daytime radiative cooling above the critical phase transition, while simultaneously providing 70% higher visual transmission as shown in Table 2. This performance enhancement is directly related to the reduced solar absorption for the metasurface structure.

In the cold phase, all devices under study provide negative radiative cooling under solar illumination. The VO_2_ thermochromic transition therefore provides the ability to switch between heating and cooling of the material depending upon its temperature. We note that these calculations do not take into account any on-board energy dissipation of the spacecraft itself which will result in additional thermal loading of the device.

Similar results are obtained for the space environment, here the effects of changes in solar absorption are even more critical and result in larger variations in net cooling power during solar irradiation between different devices. The space environment benefits from the absence of an atmospheric feedback effect, thus facilitating more effective radiation cooling. In absence of direct illumination by sunlight, the values of *P*_r_ for space and *P*_r_ − *P*_a_ for terrestrial applications show only a weak benefit from the metasurface device, indicating that the purely radiative performance is more or less the same for structured and unstructured. Here the benefit of high visual transparency remains main benefit of the metasurface device geometry.

## 5 Conclusions

In conclusion, we have demonstrated a smart IR thermal emitter with high visual transparency based on a VO_2_ metasurface, as a radiative cooling solution to spacecraft solar panels. We have optimized high IR emissivity contrast by exploring different anneals on ALD-grown VO_2_. The VO_2_ thermal emitter has demonstrated tunability of the IR emissivity Δ*ɛ* of 0.26. The metasurface device offers a 62% improvement on solar transmittance without compromising the IR emissivity tunability with respect to a planar film emitter. The improved solar transmittance of the metasurface is primarily attributed to the reduction of the VO_2_ coverage and overcomes the usual low visual transparency of VO_2_ smart films. Under daytime radiative cooling conditions both in terrestrial and space environment, the metasurface structure significantly outperforms on the equivalent thin-film devices owing to a reduction in absorbed solar power. The transparent VO_2_ thermal emitters have a thickness less than 2 µm and are compatible with integration onto a variety of devices, including solar cells, smart windows, displays, visors or glasses, enabling new opportunities for providing temperature regulating functionalities with high transparency.

## 6 Methods

***Vanadium dioxide (VO*_*2*_ *) growth by atomic layer deposition.*** The VO_2_ ALD process was carried out using Tetrakis(ethylmethylamino)vanadium(IV) (TEMAV) 98% from Strem Chemicals and deionized water as oxidizer. All films were deposited at 150 °C in a Savannah S200 ALD system. To achieve sufficient vapour pressure, TEMAV precursor was heated to 85 °C. The carrier/purge nitrogen gas was set as 20 sccm. Various dose and purge times were investigated, and the optimized parameters are as follows: TEMAV dose 0.4 s, TEMAV purge 9 s, H_2_O dose 0.1 s and H_2_O purge 12 s. For a 40 nm thick VO_2_, the growth cycle number was set to be 1100.

***Transparent metasurface emitter fabrication.*** A 300 nm Al-doped ZnO (AZO) film is deposited on 1 mm thick CaF_2_ substrates (10 × 10 mm) using an Oxford Instrument FlexAl ALD system with trimethylaluminum (TMA), diethylzinc (DEZ) and H_2_O precursors. The deposition temperature was set at 250 °C and Al doping was controlled by setting the Al/Zn cycle ratio as 4% (similar to previous work [13, 44]). Subsequently, 1200 nm silicon dioxide (SiO_2_) was deposited on the AZO film by a PECVD system. 40 nm VO_2_ was grown on the SiO_2_ using Savannah S200 ALD system and converted into VO_2_ (M1) through an anneal in O_2_ pressure of 40 mTorr and 450 °C for 1 h. Similar structures were also fabricated on SiO_2_ coated Si substrates for material characterizations. The metal oxide layer (VO_2_) was patterned by e-beam lithography using a JEOL JBX-9300FS e-beam system and Ar ion beam etch using an Oxford Instrument IonFab 300 Plus system [70]. The resist (ZEP520A) was stripped using a low temperature O_2_ ICP plasma process without further oxidizing the metal oxide surface.

### 6.1 Material characterizations

VO_2_ was characterized in a Thermo Scientific Theta Probe X-ray photoelectron spectroscopy (XPS) system in ultrahigh vacuum conditions (base pressure *P* ∼ 1 × 10^−7^ Pa). The XPS data was analyzed with Themo Avantage software. Crystallized VO_2_ (through post-deposition anneal) was characterized in a Renishaw inVia laser Raman spectrometer using 633 nm laser with the exposure power less than 1 mW to avoid film overheat [59].

### 6.2 Optical characterizations

IR reflectance was measured over the range of 1.6–20 µm using a Fourier transform infrared microscopy (FTIR, Thermo-Nicolet Nexus 670, Continuum microscope) using a ×15 optical objective and MCT-A detector. The KBr beam splitter and IR source were used and the transmittance and reflectance were normalized with air and aluminum mirror, respectively. For the visible and near-IR spectra range (0.4–1.6 µm), reflection and transmission were measured using a separate home-made using a pair of Si and InGaAs spectrometers with a supercontinuum light source (Fianium SC400-1). Two dimensional scans of the surface were performed using motorized stages (Thorlabs) to obtain spectra for the individual arrays (120 × 120 µm).

### 6.3 Numerical modeling

The simulations of the metasurface thermal emitters were done using the finite difference time domain (FDTD) method implemented in Lumerical software. Spectra from 0.4–20 µm wavelength were obtained using a broadband short pulse source. The source was a plane wave incident normal to the surface. Symmetric and antisymmetric boundary conditions were used to reduce the computation volume. The refractive index and extinction coefficients of AZO and VO_2_ as well as other materials including SiO_2_ and CaF_2_ are from tabulated references [71] and are presented in Supplementary Materials Figure S6.

## Supplementary Material

Supplementary Material Details
