# Supplementary material for: VO2 metasurface smart thermal emitter with high visual transparency for passive radiative cooling regulation in space and terrestrial applications
Source: Nanophotonics. 2022 Apr 25;11(17):4101–14. doi: 10.1515/nanoph-2022-0020 (PMC11502091; doi:10.1515/nanoph-2022-0020)
Supplement: Supplementary file 1 — Supplementary Material Details [file j_nanoph-2022-0020_suppl.docx]

**Supporting information of “VO_2_ Metasurface Smart Thermal Emitter with High Visual Transparency for Passive Radiative Cooling Regulation in Space and Terrestrial Applications”**

Kai Sun^1,2^, Wei Xiao^1,2^, Callum Wheeler^2^, Mirko Simeoni^3^, Alessandro Urbani^3^, Matteo Gaspari^3^, Sandro Mengali^3^, C.H. (Kees) de Groot^2^ and Otto L. Muskens*^,1^

^1^ Physics and Astronomy, Faculty of Physical Sciences and Engineering, University of Southampton, Southampton, SO17 1BJ, United Kingdom

^2^ Electronics and Computer Science, Faculty of Physical Sciences and Engineering, University of Southampton, Southampton SO17 1BJ, United Kingdom

^3^ Consorzio CREO, SS.17 Località Boschetto, 1-67100, L’Aquila, Italy

* corresponding author: O.Muskens@soton.ac.uk

**S1. Atomic layer deposition of VO_2_**

The TEMAV dose and purge time are plotted in Figure S1. The thickness measurements were done using Ellipsometry (0.2-1.7 µm wavelength). Figure S1a shows a consistent growth rate of about 0.036 nm/cycle for TEMAV dose time between 0.3 to 0.5 sec and the growth rate increases to above 0.04 nm/cycle at TEMAV does of 0.8 sec. Unlike the consistent growth rate over all TEMAV doses, the film uniformity (Figure 3b) shows an excellent uniformity below 3% for TEMAV doses of 0.4 and 0.5 sec, below and above which the film uniformity degrades to 5% and 3.2%, respectively. The non-uniformity at low TEMAV dose is attributed to under dose growth whilst that at high TEMAV could lead to CVD-like growth. Therefore, the TEMAV does of 0.4 sec is chosen as the optimized value for later process. Figure S1c shows the growth rate against different TEMAV purge times. The growth rate is barely affected and almost consistent in 0.036 nm/cycle for all doses. The film uniformity is 5.2% for TEMAV purge time of 6 sec and is consistent of 0.036 nm/cycle for all longer TEMAV purge times. Thus, the TEMAV purge time is optimized to be 9 sec. The optimized process gives a VO_2_ growth rate of 0.036 nm/cycle and uniformity of 1.8%.

Figure S2a shows the thickness mapping of a VO_2_ film grown by 150 cycles on a SiO_2_ coated Si substrate using the optimized condition. The TEMAV dose and purge times were set as 0.4 sec and 9 sec, respectively. The H_2_O dose and purge were set as 0.05 sec and 12 sec, respectively. The VO_2_ film was fitted to be 5.4 nm and a 6-inch uniformity of 1.8%, defined as thickness standard deviation over thickness mean. The growth rate was calculated to be 0.036 nm/cycle. Figure S2b shows the thickness as a function of ALD growth cycle number. The grown thickness is seen as a linear relation with the set ALD growth cycle number, indicating a well-controlled film growth.

(b)

(a)

(d)

(c)

Figure S1 (a) (b) Growth rate and film uniformity as a function of TEMAV dose, and (c) (d) Growth rate and Film uiformity as a function of TEMAV purge time. Solid lines are guides to the eye.

Figure S2 (a) Thickness mapping by Ellipsometry of a deposited VO_2_ film deposited on a SiO_2_ coated Si substrate and (b) thickness as a function of ALD growth cycle number.

**S2. IR Emittance hysteresis of VO_2_ film emitter**

Figure S3 shows emittance as a function of temperature through FTIR measurements. The curve shows a typical hysteresis curve with an emittance increase around 70℃ owing to the insulator to metal phase transition, consistent with our previous sputtered VO_2_ on Al reflector.^1^ IR emittance contrast is calculated to be 0.23 from difference between 30℃ and 90℃.

Figure S3 Emittance hysteresis curve of a VO_2_ film emitter. Solid lines are guides to the eye.

**S3. Effect of AZO carrier density on optical response**

Figure S4 shows simulated spectra of VO_2_ metasurfaces (3.5 µm square and 2 µm gap) with different AZO carrier densities at hot and cold states.

Figure S4 Effect of AZO carrier density by numerical simulations. (a, b, c) transmission, reflection and absorption in the hot state and (d, e, f) transmission, reflection and absorption in the cold state.

**S4. Effect of Incident Angle on optical response**

Figure S5 shows simulated optical response of VO_2_ metasurfaces (3.5 µm square and 2 µm gap) at different incident angle.

Figure S5 Effect of incident angle on optical responese by numerical simulations. (a, b, c) transmission, reflection and absorption in the hot state, (d, e, f) transmission, reflection and absorption in the cold state, extract emissivity (g), solar absorption (h) and visible transmittance (i).

**S5. Cooling power calculation**

**S5.1 Cooling power for terrestrial application**

Based on the Kirchhoff’s law, the material would also exhibit excellent thermal radiation capability as thermal dissipation. Here, we first consider the spectral radiation of a black body at temperature *T*, which can be expressed below. Blackbody radiation spectrum is given in term of wavelength:

$B\left( T,\lambda\right)=\frac{2hc^{2}}{\lambda^{5}}\frac{1}{exp\left( {hc}/{\lambda kT} \right)-1}$ *Eq.1*

where *B*(*T, λ*) is black body radiation at *T* with SI unit of *B*(*T, λ*) is W·sr^-1^·m^-3^, *h* is plank constant of 6.626×10^-34^ J.s, *c* is light speed in vacuum 3×10^8^ m/s, *k* is Boltzmann constant of 1.38×10 ^-23^ J/K and *λ* is the wavelength of the light.

**Atmosphere radiation power (*P*_a_)** can be calculated in wavelength of 2.5 to 20 µm:

$P_{a}=\int_{0}^{2\pi} d\Omega\cos\theta\int_{2.5 \mu m}^{20 \mu m} B\left( \lambda, T_{a} \right)ɛ_{s}\left( \lambda, T_{s} \right)ɛ_{a}\left( \lambda\right)$ *Eq.2*

where $\int d\Omega=2\pi\int_{0}^{\frac{\pi}{2}} sin\theta d\theta$ is the angular integral over a hemisphere from Stefan-Boltzmann law*, T_a_* is ambient temperature (*T*_a_) assumed to be 300 K, $ɛ_{a}\left( \lambda\right)$ is the absorptivity of the atmosphere^2^ and $ɛ_{s}\left( \lambda, T_{s} \right)$ is the absorptivity of the emitter, which is calculated from FTIR measurements. The emitter surface temperature (*T_s_*) is assumed to be 90℃ and 30℃ in case of Hot and Cold status, respectively.

**Emitter blackbody radiation power (*P*_r_)** can be calculated in wavelength of 2.5 to 20 µm:

$P_{r}=\int_{0}^{2\pi} d\Omega\cos\theta\int_{2.5 \mu m}^{20 \mu m} B\left( \lambda,T_{s} \right)ɛ_{s}\left( \lambda, T_{s} \right)d\lambda$ *Eq.3*

For the emitter, the temperature (*T_s_*) is assumed to be 90℃ and 30℃ in case of Hot and Cold status, respectively.

**Sun radiation absorption power (*P*_sun_)** using experimental radiation spectrum (I_AM1.5_), wavelength 0.4 to 2.5 um. The I_AM1.5_ is solar radiation spectrum after through the atmosphere for terrestrial case, which is available from the website.^3^ Thus, the sun radiation absorption power on the earth is calculated as:

$P_{sun}=\int_{0.4 \mu m}^{2.5 \mu m} I_{AM1.5}\left( \lambda,T_{s} \right)ɛ_{s}\left( \lambda, T_{s} \right)d\lambda$ *Eq.4*

**S5.2 Cooling power for space application**

For space application, there is no atmosphere influence. The emitter IR radiation (*P_r_*) is unchanged and can be calculated from Eq. 3. For solar radiation above atmosphere, the solar radiation absorption is calculated as:

$P_{sun}=\frac{r^{2}}{R^{2}}\int_{0}^{2\pi} d\Omega\cos\theta\int_{0.4 \mu m}^{2.5 \mu m} B(T=5777K,\lambda)\varepsilon_{s}\left( \lambda\right)d\lambda$ *Eq.5*

where *R* is the mean distance between the earth and the sun and *r* is the mean radius of the sun.

**S6. Optical properties of materials for simulations.**

Figure S6 shows refractive index used in numerical simulations, with VO_2_ and SiO_2_ optical properties from literature works.^4-5^

Figure S6 Refractive index of VO_2_, AZO (from ellipsometry) and SiO_2_ used in the numerical simulations, (a) n and (b) k.

Uncategorized References

1. Sun, K.; Riedel, C. A.; Urbani, A.; Simeoni, M.; Mengali, S.; Zalkovskij, M.; Bilenberg, B.; de Groot, C. H.; Muskens, O. L., VO2 Thermochromic Metamaterial-Based Smart Optical Solar Reflector. *ACS Photonics* **2018,** *5* (6), 2280-2286.

2. Atmospheric Transmission Data. <https://webarchive.gemini.edu/20210519-sciops--instruments--mid-ir-resources--spectroscopic-calibrations/atmospheric-transmission-data.html>.

3. Standard Solar Spectra. <https://www.pveducation.org/pvcdrom/appendices/standard-solar-spectra>.

4. Kitamura, R.; Pilon, L.; Jonasz, M., Optical constants of silica glass from extreme ultraviolet to far infrared at near room temperature. *Applied Optics* **2007,** *46* (33), 8118-8133.

5. Verleur, H. W.; Barker, A. S.; Berglund, C. N., Optical Properties of VO_2_ between 0.25 and 5 eV. *Physical Review* **1968,** *172* (3), 788-798.
